# Mitochondrial-Targeting Antioxidant SS-31 Suppresses Airway Inflammation and Oxidative Stress Induced by Cigarette Smoke

**DOI:** 10.1155/2021/6644238

**Published:** 2021-06-15

**Authors:** De-qing Yang, Qiu-nan Zuo, Tao Wang, Dan Xu, Liu Lian, Li-juan Gao, Chun Wan, Lei Chen, Fu-qiang Wen, Yong-chun Shen

**Affiliations:** ^1^Division of Pulmonary Diseases, State Key Laboratory of Biotherapy of China and Department of Respiratory and Critical Care Medicine, West China Hospital of Sichuan University, Chengdu 610041, China; ^2^Respiratory Ward, Department of Geriatrics, Sichuan Provincial People's Hospital, University of Electronic Science and Technology of China, Chengdu 610072, China

## Abstract

This study investigated whether the mitochondrial-targeted peptide SS-31 can protect against cigarette smoke- (CS-) induced airway inflammation and oxidative stress *in vitro* and *in vivo*. Mice were exposed to CS for 4 weeks to establish a CS-induced airway inflammation model, and those in the experimental group were pretreated with SS-31 1 h before CS exposure. Pathologic changes and oxidative stress in lung tissue, inflammatory cell counts, and proinflammatory cytokine levels in bronchoalveolar lavage fluid (BALF) were examined. The mechanistic basis for the effects of SS-31 on CS extract- (CSE-) induced airway inflammation and oxidative stress was investigated using BEAS-2B bronchial epithelial cells and by RNA sequencing and western blot analysis of lung tissues. SS-31 attenuated CS-induced inflammatory injury of the airway and reduced total cell, neutrophil, and macrophage counts and tumor necrosis factor- (TNF-) *α*, interleukin- (IL-) 6, and matrix metalloproteinase (MMP) 9 levels in BALF. SS-31 also attenuated CS-induced oxidative stress by decreasing malondialdehyde (MDA) and myeloperoxidase (MPO) activities and increasing that of superoxide dismutase (SOD). It also reversed CS-induced changes in the expression of mitochondrial fission protein (MFF) and optic atrophy (OPA) 1 and reduced the amount of cytochrome c released into the cytosol. Pretreatment with SS-31 normalized TNF-*α*, IL-6, and MMP9 expression, MDA and SOD activities, and ROS generation in CSE-treated BEAS-2B cells and reversed the changes in MFF and OPA1 expression. RNA sequencing and western blot analysis showed that SS-31 inhibited CS-induced activation of the mitogen-activated protein kinase (MAPK) signaling pathway *in vitro* and *in vivo*. Thus, SS-31 alleviates CS-induced airway inflammation and oxidative stress via modulation of mitochondrial function and regulation of MAPK signaling and thus has therapeutic potential for the treatment of airway disorders caused by smoking.

## 1. Introduction

Cigarette smoke (CS) contains thousands of toxins and is one of the most important risk factors for the development of chronic obstructive pulmonary disease (COPD), a progressive lung condition characterized by persistent airway inflammation and irreversible restriction of airflow [1, 2]. COPD is a major health concern worldwide because of its high morbidity, mortality, and associated healthcare costs [3, 4]. The pathogenesis of COPD is complex and is not fully understood. CS induces chronic airway inflammation, airway mucus hypersecretion, and oxidative stress, leading to clinically significant mechanical obstruction of small airways, reduced airflow, and a progressive decline in lung function [5, 6].

Mitochondria are the organelles responsible for energy metabolism and play an important role in maintaining cell function. Recent studies have suggested that CS can cause mitochondrial dysfunction and trigger inflammatory responses and oxidative stress, which are linked to COPD [7, 8]. SS-31, a novel mitochondrial-targeting antioxidant compound, can eliminate reactive oxygen species (ROS) and increase ATP production in mitochondria, thus restoring mitochondrial membrane potential [9]. SS-31 was shown to protect cultured mouse microglial cells against lipopolysaccharide-induced inflammation and oxidative stress by stabilizing mitochondrial structure and reducing mitochondrial fission (FIS) 1 protein expression [10]. It also alleviated the inflammatory response and oxidative stress and exerted beneficial effects on leukocytes in type 2 diabetes patients [11]. In a mouse model of spinal cord injury-induced lung impairment, SS-31 attenuated mitochondrial dysfunction and inflammation and reduced the severity of lung damage [12]. However, it is unclear whether SS-31 can protect against CS-induced airway inflammation and oxidative stress.

In this study, we investigated the effect of SS-31 on inflammation and oxidative stress in the lung induced by CS *in vitro* and *in vivo* along with the underlying molecular mechanisms.

## 2. Materials and Methods

### 2.1. Animals

Male C57BL/6J mice (age 9–10 weeks, weighing 20–22 g) bred under specific pathogen-free conditions were purchased from GemPharmatech (Nanjing, Jiangsu, China) and housed at constant temperature of 23°C ± 2°C and 50% ± 10% humidity on a 12 : 12 h light/dark cycle (lights on from 6:00 a.m. to 6:00 p.m.). The mice had free access to food and water. Experimental procedures were conducted under aseptic conditions. Chambers and cages were cleaned every 3 days. The mice were handled in accordance with the ARRIVE guidelines developed by the National Center for the Replacement, Refinement, and Reduction of Animals in Research. The study protocol was reviewed and approved by the Animal Ethics Committee of West China Hospital, Sichuan University (approval no. 2020229A).

### 2.2. CS Exposure and Animal Treatment

Mice were divided into 4 groups: a control group (*n* = 7) exposed to room air without treatment, and 3 experimental groups exposed to CS for 75 min twice daily, 5 days per week for 4 weeks [13]. One experimental group (CS group, *n* = 7) received no treatment; the SS-31 (L)+CS group (*n* = 8) was exposed to CS and treated with a low dose of SS-31 (2.5 mg/kg once daily) refer to the slightly modified dosage [14]; and the SS-31 (H)+CS group (*n* = 7) was exposed to CS and treated with a high dose of SS-31 (5 mg/kg once daily) [15, 16]. SS-31 (Topscience, Shanghai, China) was intraperitoneally injected 1 h before CS exposure [13, 17]. After 4 weeks, all mice were sacrificed by intraperitoneal phenobarbital injection (Sigma-Aldrich, St. Louis, MO, USA) followed by exsanguination from the right ventricle and abdominal aorta. The heart was flushed with 10 mL sterile phosphate-buffered saline (PBS) from the right ventricle until the lungs turned white to collect bronchoalveolar lavage fluid (BALF) and lung tissue samples.

### 2.3. BALF Collection and Cell Counting

Right lungs were washed 3 times with 0.5 mL of sterile PBS, and >1.3 mL of BALF was recovered from each mouse and centrifuged at 1000×*g* for 5 min. The supernatant was stored at −80°C for analysis of cytokine levels by enzyme-linked immunosorbent assay (ELISA). The cell pellet was resuspended in 0.2 mL PBS, and the total cell number was counted with a hemocytometer. Differential cell counting was performed by cytocentrifugation (Cytopro7620; Wescor, UT, USA) at 100×*g* for 10 min followed by Wright–Giemsa staining.

### 2.4. Histologic Examination of Lung Tissue

Left lungs without lavage were fixed with 4% phosphate-buffered paraformaldehyde under a constant pressure of 25 cm H_2_O and embedded in paraffin; 4 mm thick sections were cut and stained with hematoxylin and eosin. An experienced pathologist who was blinded to the treatments scored lung inflammation based on the severity of lung lesions including peribronchiolar infiltrates, alveolar septal infiltrates, perivascular infiltrates, and combined bronchus-associated lymphoid tissue hyperplasia [13]. Giemsa staining was performed to assess the density of inflammatory cells in alveoli.

Alcian blue- (AB-) periodic acid Schiff (PAS) staining was performed to assess the levels of intracellular mucous glycoconjugates. Immunohistochemical staining for Muc5ac protein was performed using a kit (Santa Cruz Biotechnology, Santa Cruz, CA, USA). Briefly, lung sections were stained with anti-Muc5ac antibody (clone 45 M1, 1 : 100; Genetex, Irvine, CA, USA) and the percentage of the total airway epithelial area that was positive for AB/PAS staining or anti-Muc5ac immunoreactivity was quantified using Image-Pro Plus v6.0 software (Media Cybernetics, Bethesda, MD, USA).

### 2.5. CS Extract (CSE) Preparation

CSE was freshly prepared as previously described [18], with a few modifications. Briefly, the smoke of 6 Marlboro cigarettes was bubbled through 20 mL of Dulbecco's modified Eagle's medium (DMEM) prewarmed at 37°C. The solution was passed through a 0.22 *μ*m filter after adjusting the pH to 7.4, yielding 100% CSE. Serum-free DMEM was used to dilute the 100% CSE to the working concentrations.

### 2.6. Cell Culture and Treatments

BEAS-2B normal human bronchial epithelial cells (American Type Culture Collection, Manassas, VA, USA) were cultured in DMEM supplemented with 10% fetal bovine serum and 1% penicillin G sodium/streptomycin sulfate (Invitrogen, Carlsbad, CA, USA). The effect of CSE on cell viability was evaluated with the Cell Counting Kit- (CCK-) 8 assay (Dojindo Laboratories, Tokyo, Japan). Cells (5 × 10^3^/well) were seeded in 96-well plates and allowed to attach overnight, then incubated for 24 h in DMEM containing CSE (2%, 4%, 6%, 8%, and 10%). After removing the supernatant, 10 *μ*L of CCK-8 reagent in a serum-free medium (100 *μ*L/well) was added, followed by incubation for 1 h at 37°C. Absorbance was measured on a spectrophotometer at 450 nm.

To evaluate the role of the mitogen-activated protein kinase (MAPK) signaling pathway in the anti-inflammatory and antioxidant effects of SS-31, BEAS-2B cells were pretreated with the MAPK activator anisomycin (20 *μ*g/mL/mL) (APExBIO Technology, Houston, TX, USA) for 1 h [19]. The cells were divided into 5 groups: control, CSE (incubation with CSE for 24 h), anisomycin+CSE (incubation with CSE for 24 h following pretreatment with 20 *μ*g/mL anisomycin for 1 h), anisomycin+SS-31+CSE group (treatment with 100 *μ*M SS-31 for 1 h followed by CSE for 24 h after pretreatment with 20 *μ*g/mL anisomycin for 1 h), and SS-31+CSE (incubation with CSE for 24 h following pretreatment with 100 *μ*M SS-31 for 1 h). Cells or the supernatants were harvested for analyses. The dose of SS-31 and incubation time of CSE were selected based on previous studies [20–22].

### 2.7. Measurement of Inflammatory Cytokine Levels

BALF levels of interleukin- (IL-) 6 and tumor necrosis factor- (TNF-) *α* were measured with ELISA kits for mice (NeoBioscience, Shenzhen, China) according to the manufacturers' instructions. The level of matrix metallopeptidase (MMP) 9 was measured with an ELISA kit for mice (R&D Systems, Minneapolis, MN, USA) according to the manufacturer's instructions; the stated detection limits were 15 pg·mL^−1^ for TNF-*α*, 1.6 pg·mL^−1^ for IL-6, and 0.014 ng·mL^−1^ for MMP9. TNF-*α*, IL-6, and MMP9 levels in culture supernatant were determined with ELISA kits for humans (NeoBioscience) according to the manufacturers' instructions; the stated detection limits were 7.8 pg·mL^−1^ for TNF-*α*, 0.39 pg·mL^−1^ for IL-6, and 15.6 pg·mL^−1^ for MMP9.

### 2.8. Detection of Oxidative Stress and Intracellular ROS

Malondialdehyde (MDA), superoxide dismutase (SOD), myeloperoxidase (MPO) activities in mouse lung homogenate and glutathione peroxidase (GSH-Px), MDA, and SOD activities as well as intracellular ROS production in cells were determined according to commercial protocols (Nanjing Jiancheng Bioengineering Institute, Jiangsu, China).

### 2.9. RNA Sequencing

RNA sequencing was performed as previously described [23]. Briefly, total RNA from lung tissue of the CS and SS-31 (H)+CS groups was isolated and the quality was verified according to the protocols of Illumina (San Diego, CA, USA). A total of 2 *μ*g RNA per sample was used as input material for library construction. Strand-specific sequencing libraries were generated by the dUTP method with RNA obtained using the NEBNext Ultra Directional RNA Library Prep Kit (Illumina) according to the manufacturer's instructions. RNA sequencing was performed on an Illumina Hiseq 2000 platform by Genewiz (Suzhou, China), generating 100 bp paired-end reads. Adapter sequences were removed from the raw sequencing data, and the individual libraries were converted to FASTQ format. Sequence reads were aligned to the mouse genome (mm10) with TopHat2 v2.0.9, and the resultant alignment files were reconstructed with Cufflinks v2.1.1 and Scripture (beta2). For mRNA analyses, the RefSeq database (Build 37.3) was used as the source of annotation references. The read counts of each transcript were normalized to the length of the individual transcript and to the total mapped fragment counts in each sample and expressed as fragments per kilobase of exon per million fragments of mRNA mapped in each sample. Differential expression analyses were conducted using only samples from the CS and SS-31 (H)+CS groups. *P* < 0.05 was used as the cutoff for differentially expressed genes (DEGs).

### 2.10. Bioinformatic Analysis

Enrichment analysis of DEGs was carried out to detect overrepresented functional terms in the genomic background. Gene Ontology (GO) analysis of biological processes, cellular components, and molecular function was performed using the GO-seq R package [24]. Enriched DEG signaling pathway analyses were conducted using the Kyoto Encyclopedia of Genes and Genomes (KEGG) database.

### 2.11. Western Blot

Protein samples were isolated from the right lung of mice and from BEAS-2B cells with radioimmunoprecipitation assay lysis buffer supplemented with 1 mM phenylmethanesulfonyl fluoride (Cell Signaling Technology, Danvers, MA, USA). Total protein was fractionated by 10% sodium dodecyl sulfate-polyacrylamide gel electrophoresis and transferred to a polyvinylidene difluoride membrane. After blocking with 5% bovine serum albumin in Tris-buffered saline at room temperature for 1 h, the membrane was incubated overnight at 4°C with antibodies against optic atrophy (OPA) 1 (mouse antibody from Cell Signaling Technology; human antibody from Proteintech, Rosemont, IL, USA); mitochondrial fission factor (MFF) (mouse antibody from Cell Signaling Technology; human antibody from Proteintech); and extracellular signal-regulated kinase (ERK), phosphorylated- (P-) ERK, P38, P-P38, and glyceraldehyde 3-phosphate dehydrogenase (GAPDH) (all from Cell Signaling Technology). They were then incubated with horseradish peroxidase-conjugated secondary antibodies, and immune complexes were detected with SuperSignal West Pico chemiluminescent substrate (Pierce, Rockford, IL, USA).

Cytoplasmic proteins were extracted using the Cytoplasmic Protein Extraction Kit (KeyGEN BioTech, Nanjing, China), and mitochondrial proteins were extracted using the Mitochondria Isolation Kit (Beyotime Biotech, Shanghai, China). Cytoplasmic extracts were probed with antibodies against cytosolic cytochrome c (Genetex) and GAPDH (Cell Signaling Technology). Mitochondrial extracts were probed with antibodies against mitochondrial cytochrome c (Genetex) and cytochrome c oxidase (COX) IV (Proteintech). Each experiment was repeated 3 times with different mice. The signal intensity of protein bands was quantified using ImageJ software (National Institutes of Health, Bethesda, MD, USA).

### 2.12. Statistical Analysis

Data are expressed as mean ± standard deviation, and group means were compared by one-way analysis of variance followed by the least significant difference test for multiple comparisons. Data were analyzed, and figures were prepared using Prism 7 software (GraphPad, San Diego, CA, USA). *P* < 0.05 was considered statistically significant.

## 3. Results

### 3.1. SS-31 Reverses the CS-Induced Increases in Inflammatory Cell Numbers and Cytokine Release in Mouse Lung

**Figure 1:**
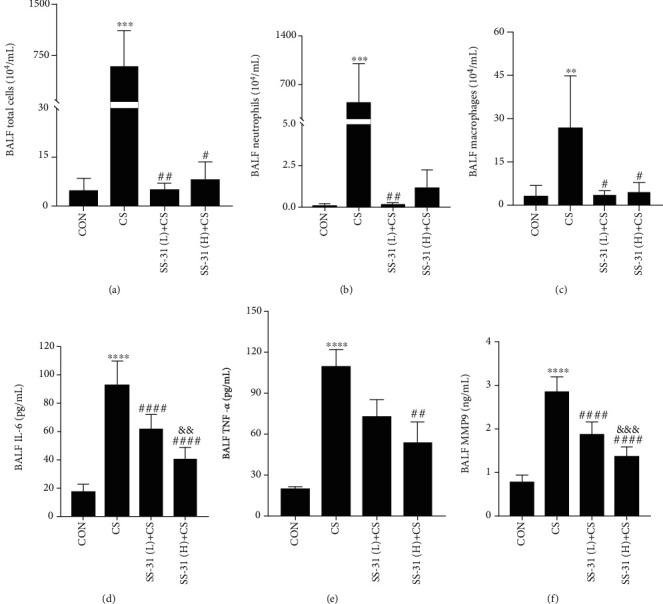
SS-31 reduces CS-induced increases in inflammatory cell numbers and cytokine infiltration in mouse lung. (a–f) Total cell (a), neutrophil (b), and macrophage (c) counts and IL-6 (d), TNF-*α* (e), and MMP9 (f) levels in mouse BALF (control: *n* = 7, CS: *n* = 5, SS-31 (L)+CS: *n* = 8, SS-31 (H)+CS: *n* = 5). ^∗∗^*P* < 0.01, ^∗∗∗^*P* < 0.001, and ^∗∗∗∗^*P* < 0.0001 vs. control; ^#^*P* < 0.05, ^##^*P* < 0.01, and ^####^*P* < 0.0001 vs. CS; ^&&^*P* < 0.01, ^&&&^*P* < 0.001, and SS-31 (L)+CS vs. SS-31 (H)+CS. Abbreviations: BALF: bronchoalveolar lavage fluid; CS: cigarette smoke; SS-31 (H): high-dose SS-31 (5 mg/kg); SS-31 (L): low-dose SS-31 (2.5 mg/kg).

Total cell, neutrophil, and macrophage counts in mouse BALF were increased in CS-exposed mice, which was abrogated by pretreatment with SS-31 (Figures 1(a)–1(c)). Meanwhile, 4 weeks of CS exposure increased the levels of IL-6, TNF-*α*, and MMP9 in BALF, an effect that was abolished by SS-31 (Figures 1(d)–1(f)).

### 3.2. SS-31 Abrogates Histologic Changes in the Lung Induced by CS

**Figure 2:**
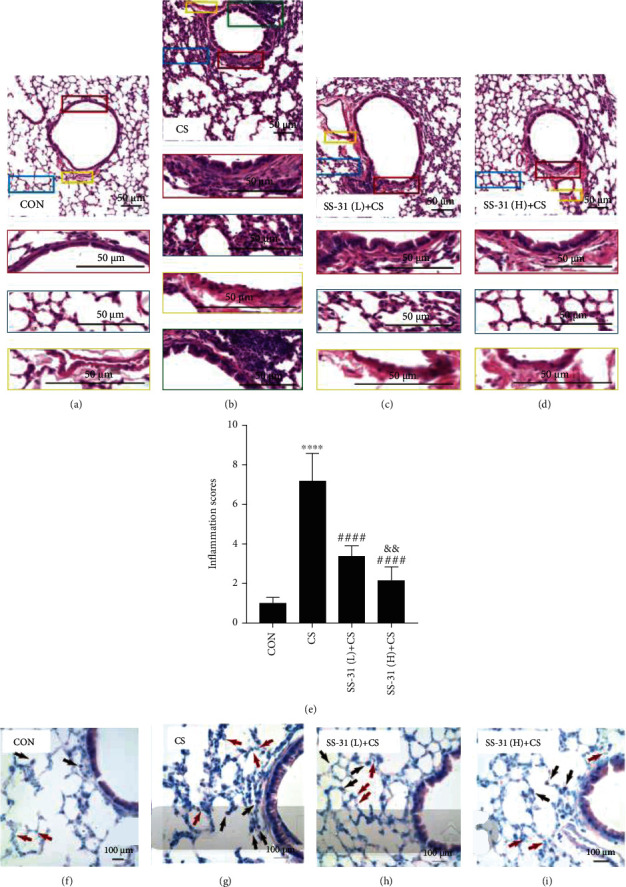
SS-31 attenuates CS-induced histologic changes in mouse lung. (a–d) H&E staining of mouse lung tissue from control (*n* = 7) (a), CS (*n* = 7) (b), SS-31 (L)+CS (*n* = 8) (c), and SS-31 (H)+CS (*n* = 7) (d) groups (200x magnification, scale bar = 50 *μ*m). (e) Inflammation scores of mouse lungs. Bottom images depict an enlarged view of boxed areas in corresponding top images; red box: peribronchiolar infiltrates; blue box: alveolar septal infiltrates; yellow box: perivascular infiltrates; green box: bronchus-associated lymphoid tissue hyperplasia. (f–i) Giemsa staining of mouse lung tissue from control (f), CS (g), SS-31 (L)+CS (h), and SS-31 (H)+CS (i) groups (600x magnification, scale bar = 100 *μ*m). Red arrow: neutrophils; black arrow: macrophages. ^∗∗∗∗^*P* < 0.0001 vs. control; ^####^*P* < 0.0001 vs. CS; ^&&^*P* < 0.01 SS-31 (L)+CS vs. SS-31 (H)+CS. Abbreviations: CS: cigarette smoke; H&E: hematoxylin and eosin; SS-31 (H): high-dose SS-31 (5 mg/kg); SS-31 (L): low-dose SS-31 (2.5 mg/kg).

Four weeks of exposure to CS markedly increased peribronchial inflammatory cell infiltration, airway epithelial cell hyperplasia, airway epithelium thickening, and lumen obstruction by mucus and cell debris (Figures 2(b) and 2(e)). These changes were abolished by low-dose (2.5 mg/kg) and high-dose (5 mg/kg) SS-31 pretreatment, with the latter yielding better results (*P* < 0.01; Figure 2(e)). Giemsa staining showed that the number of neutrophils and macrophages was increased in CS-exposed mice, but this was reversed by SS-31 pretreatment (Figures 2(f)–2(i)).

### 3.3. SS-31 Suppresses CS-Induced Airway Mucus Hypersecretion

**Figure 3:**
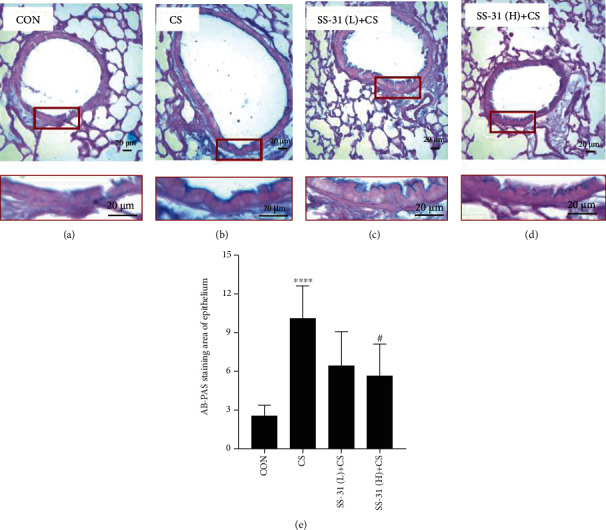
SS-31 attenuates CS-induced airway mucus hypersecretion. (a–d) Mouse lung tissue from control (*n* = 7) (a), CS (*n* = 7) (b), SS-31 (L)+CS (*n* = 8) (c), and SS-31 (H)+CS (*n* = 7) (d) groups after AB/PAS staining (400x magnification, scale bar = 20 *μ*m). (e) The positive percentage of epithelial area with AB/PAS staining. Bottom images depict enlarged views of boxed areas in corresponding top images. ^∗∗∗∗^*P* < 0.0001 vs. control; ^#^*P* < 0.05 vs. CS. Abbreviations: AB/PAS: Alcian blue/periodic acid Schiff; CS: cigarette smoke; SS-31 (H): high-dose SS-31 (5 mg/kg); SS-31 (L): low-dose SS-31 (2.5 mg/kg).

**Figure 4:**
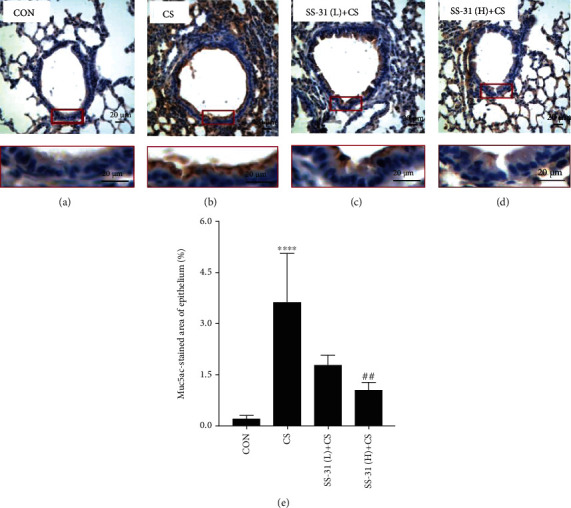
SS-31 suppresses CS-induced airway mucus protein secretion. (a–d) Immunohistochemical detection of Muc5ac in mouse airway epithelium (400x magnification, scale bar = 20 *μ*m) from control (a), CS (b), SS-31 (L)+CS (c), and SS-31 (H)+CS (d) groups (*n* = 7 per group). (e) The positive percentage of epithelial area with anti-Muc5ac immunoreactivity. Bottom images depict enlarged views of boxed areas in corresponding top images. ^∗∗∗∗^*P* < 0.0001 vs. control; ^##^*P* < 0.01 vs. CS. Abbreviations: CS: cigarette smoke; SS-31 (H): high-dose SS-31 (5 mg/kg); SS-31 (L): low-dose SS-31 (2.5 mg/kg).

Mucus proteins were stained with AB/PAS. CS exposure significantly increased the secretion of airway mucus proteins (Figure 3), but this was suppressed by high-dose SS-31 pretreatment. Accordingly, the airway mucus protein Muc5ac was upregulated after 4 weeks of CS exposure (Figures 4(b) and 4(e)) compared to the control group (Figure 4(a)), but this was abrogated by high-dose SS-31 (Figures 4(d) and 4(e)).

### 3.4. SS-31 Attenuates CS-Induced Oxidative Stress in Mouse Lung

**Figure 5:**
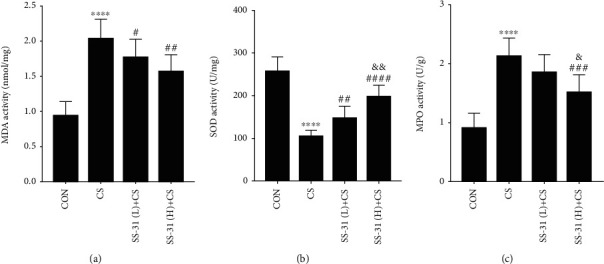
SS-31 attenuates CS-induced oxidative stress in mouse lung. (a–c) Effects of SS-31 on lung MDA (a), SOD (b), and MPO (c) activities in CS-exposed mice (*n* = 7 per group). ^∗∗∗∗^*P* < 0.0001 vs. control; ^#^*P* < 0.05, ^##^*P* < 0.01, ^###^*P* < 0.001, and ^####^*P* < 0.0001 vs. CS; ^&^*P* < 0.05, ^&&^*P* < 0.01, and SS-31 (L)+CS vs. SS-31 (H)+CS. Abbreviations: CS: cigarette smoke; MDA: malondialdehyde; MPO: myeloperoxidase; SOD: superoxide dismutase; SS-31 (H): high-dose SS-31 (5 mg/kg); SS-31 (L): low-dose SS-31 (2.5 mg/kg).

After 4 weeks of CS exposure, MDA and MPO activities were increased (Figures 5(a) and 5(c)) whereas SOD activity was decreased (Figure 5(b)) in the lungs of mice, indicating that oxidative stress was induced. SS-31 pretreatment mitigated this effect in a dose-dependent manner (Figure 5): SOD and MPO activities were significantly higher in mice pretreated with 5 mg/kg SS-31 as compared to 2.5 mg/kg SS-31 before CS (Figures 5(b) and 5(c)), suggesting that SS-31 has strong antioxidant activity. MPO activity in the lungs was positively correlated with neutrophil count (Supplementary Figure 1).

### 3.5. SS-31 Protects against Mitochondrial Dysfunction in CS-Exposed Mouse Lung

**Figure 6:**
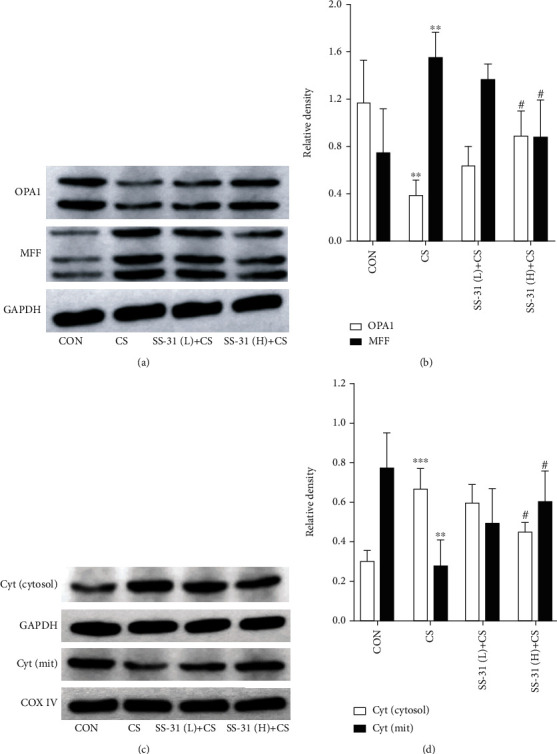
SS-31 prevents mitochondrial dysfunction in CS-induced mouse lung. (a–d) Protein levels of OPA1, MFF (a, b), and cytochrome c (c, d) in lungs were evaluated by western blot following exposure to CS with or without SS-31 pretreatment. GAPDH or COX IV was used as a loading control (*n* = 3 per group). ^∗∗^*P* < 0.01 and ^∗∗∗^*P* < 0.001 vs. control; ^#^*P* < 0.05 vs CS. Abbreviations: COX IV: cytochrome c oxidase IV; CS: cigarette smoke; Cyt c: cytochrome c; GAPDH: glyceraldehyde 3-phosphate dehydrogenase; MFF: mitochondrial fission factor; mit: mitochondrion; OPA1: optic atrophy 1; SS-31 (H): high-dose SS-31 (5 mg/kg); SS-31 (L): low-dose SS-31 (2.5 mg/kg).

The levels of the mitochondrial fusion protein OPA1 and fission protein MFF were assayed as markers of mitochondrial function. CS exposure for 4 weeks reduced the expression of OPA1 and increased that of MFF in the lungs (Figures 6(a) and 6(b)). This was accompanied by a decrease in mitochondrial cytochrome c but an increase in the cytosolic pool (Figures 6(c) and 6(d)), which was reversed by high-dose SS-31 pretreatment. The western blot analysis and densitometry results are summarized in Supplementary Table 1.

### 3.6. RNA Sequencing Analysis of Mouse Lung

**Figure 7:**
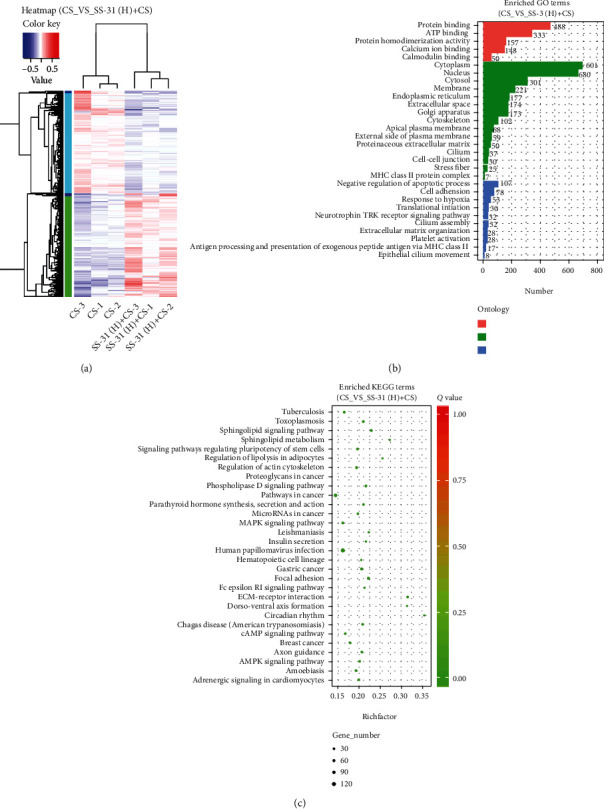
RNA sequencing in mouse lung. (a) Clustering of DEGs in the CS and SS-31 (H)+CS groups (*n* = 3 per group). (b) GO analysis of DEGs. The figure is composed of 3 parts: molecular functions, cellular components, and biological processes. The significance level of enrichment was set as a corrected *P* value < 0.05. (c) KEGG pathway analysis of DEGs. The bubble chart shows enrichment of DEGs in signaling pathways. The *y*-axis label represents the pathway, and the *x*-axis label represents the enrichment factor—i.e., the number of DEGs enriched in the pathway divided by the total number of genes in the background gene set. The size of each bubble represents the number of DEGs enriched in the pathway, and the color represents the significance of enrichment. Abbreviations: CS: cigarette smoke; DEG: differentially expressed gene; GO: Gene Ontology; KEGG: Kyoto Encyclopedia of Genes and Genomes; SS-31 (H): high-dose SS-31 (5 mg/kg).

DEGs were defined as genes with a fold change ≥ 1.0 in the SS-31 (H)+CS group compared to the CS group. According to this criterion, 4038 DEGs were identified (Figure 7(a)) including 2034 upregulated and 2004 downregulated genes; the top 10 of each are summarized in Supplementary Table 2. The DEGs were categorized into 30 GO categories under 3 ontologies (Figure 7(b)) and 30 KEGG pathways (Figure 7(c)). The KEGG pathway analysis revealed that these genes were enriched in the MAPK, cyclic (c) AMP, and 5′ AMP-activated protein kinase (AMPK) signaling pathways and extracellular matrix-receptor interaction (Figure 7(c)).

### 3.7. SS-31 Inhibits CS-Induced Phosphorylation of ERK and P38

**Figure 8:**
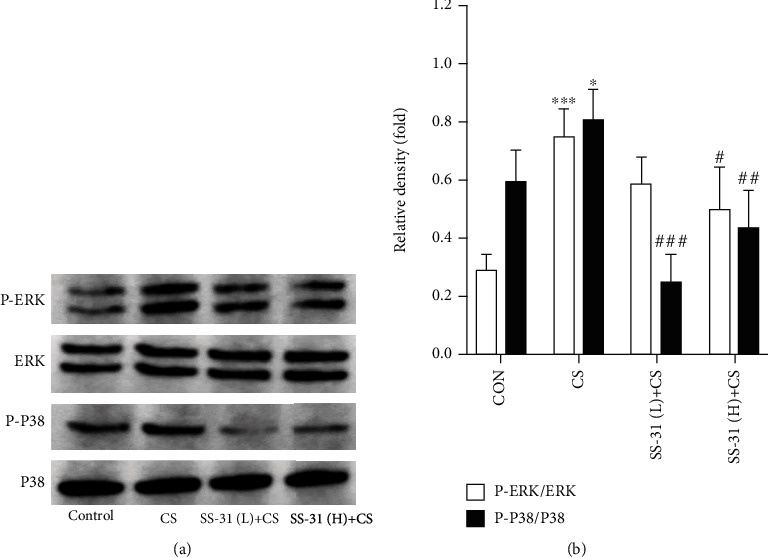
SS-31 alleviates CS-induced phosphorylation of ERK and P38. (a) Protein levels of ERK, P-ERK, P38, and P-P38 were detected by western blot. (b) Activation of these signaling molecules was evaluated based on the ratio of the density of phosphorylated protein to total protein (*n* = 3 per group). ^∗^*P* < 0.05 and ^∗∗∗^*P* < 0.001 vs. control; ^#^*P* < 0.05, ^##^*P* < 0.01, and ^###^*P* < 0.001 vs. CS. Abbreviations: CS: cigarette smoke; ERK: extracellular signal-regulated kinase; P-ERK: phosphorylated extracellular signal-regulated kinase; P-P38: phosphorylated P38; SS-31 (H): high-dose SS-31 (5 mg/kg); SS-31 (L): low-dose SS-31 (2.5 mg/kg).

The signaling pathway potentially mediating the effects of SS-31 was investigated by evaluating the expression of proteins in mouse lung tissue samples by western blot. After 4 weeks of CS exposure, the phosphorylation of ERK and P38 was increased in mouse lung. High-dose SS-31 abrogated the activation of both proteins (Figure 8). The western blot analysis and densitometry results are summarized in Supplementary Table 1.

### 3.8. SS-31 Prevents CSE-Induced Inflammatory Cytokine Release via Inactivation of MAPK Signaling in Bronchial Epithelial Cells

**Figure 9:**
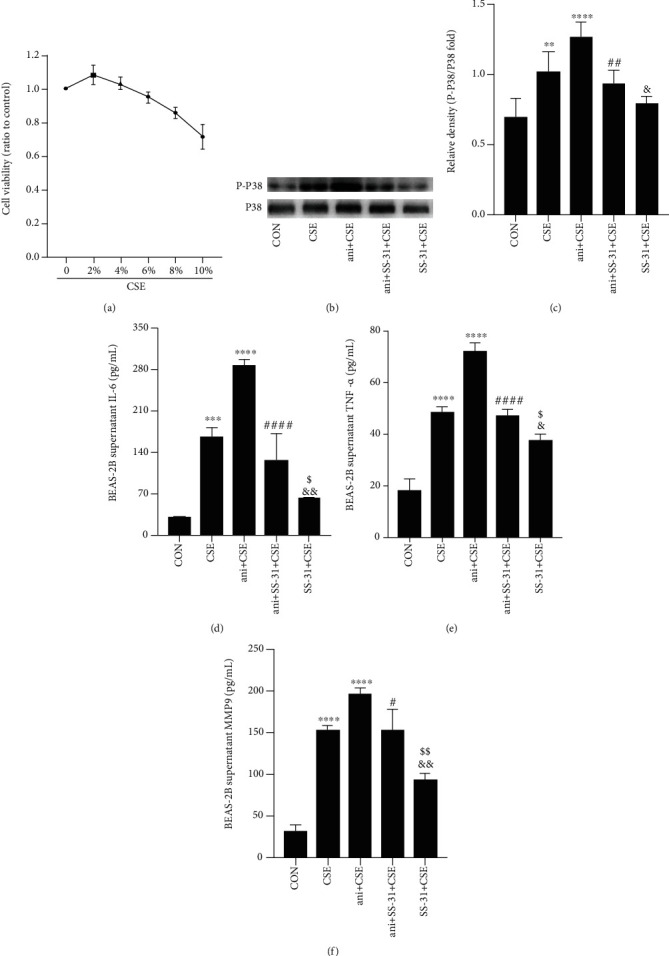
SS-31 inhibits CSE-induced inflammation and activation of MAPK signaling in BEAS-2B cells. (a) Cell viability was measured with CCK-8 following treatment with indicated concentrations of CSE (*n* = 3 per group). (b, c) Phosphorylated and total P38 protein levels (*n* = 3 per group). (d–f) IL-6 (d), TNF-*α* (e), and MMP9 (f) levels in BEAS-2B cell supernatant (*n* = 3 per group). ^∗∗^*P* < 0.01, ^∗∗∗^*P* < 0.001, and ^∗∗∗∗^*P* < 0.0001 vs. control; ^#^*P* < 0.05, ^##^*P* < 0.01, and ^####^*P* < 0.0001 vs. ani+CSE; ^&^*P* < 0.05 and ^&&^*P* < 0.01 vs CSE; ^$^*P* < 0.05, ^$$^*P* < 0.01, and ani+SS-31+CSE vs. SS-31+CSE. Abbreviations: ani: anisomycin; CSE: cigarette smoke extract; P-P38: phosphorylated P38.

To confirm the *in vivo* finding that SS-31 attenuated CS-induced inflammation and oxidative stress via inhibition of P38 MAPK signaling and to more closely examine the underlying mechanism, we evaluated the effects of anisomycin on BEAS-2B cells pretreated with CSE and SS-31. We first assessed the toxicity of CSE with the CCK8 assay. The cell viability in cells treated with 2%, 4%, 6%, and 8% CSE was >80% compared to the control group, but 10% CSE considerably decreased the percentage of living cells (<80%) (Figure 9(a)). To establish a bronchial epithelial cell model of CSE-induced airway inflammation, BEAS-2B cells were treated with 8% CSE for 24 h in the following experiments.

The P38 MAPK activator anisomycin was used to stabilize P38 at the protein level. Anisomycin significantly increased P38 phosphorylation (Figure 9(b)). SS-31 reduced the level of phosphorylated P38 MAPK in CSE-incubated BEAS-2B cells, but this was blocked by anisomycin (Figures 9(b) and 9(c)). Moreover, the levels of IL-6, TNF-*α*, and MMP9 were higher in the anisomycin+CSE+SS-31 group than in the SS-31+CSE group (Figures 9(d)–9(f)). These results imply that SS-31 inhibits MAPK signaling to alleviate CSE-induced inflammation.

### 3.9. SS-31 Prevents CSE-Induced Oxidative Stress via Inactivation of MAPK Signaling

**Figure 10:**
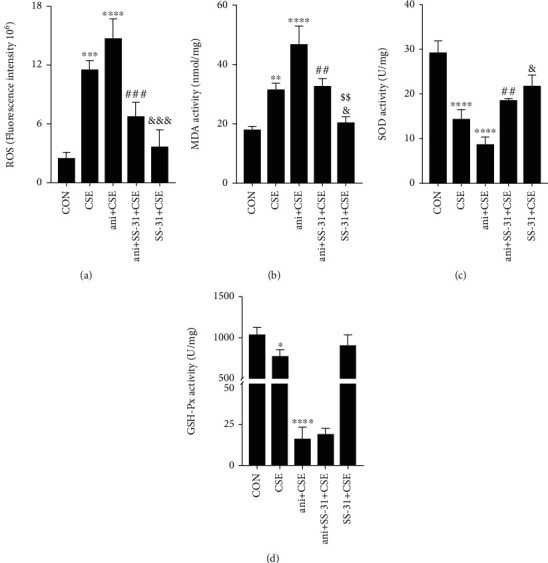
SS-31 suppresses CSE-induced oxidative stress via inhibition of MAPK signaling. (a) Intracellular ROS generation in BEAS-2B cells. (b–d) Activities of MDA (b), SOD (c), and GSH-Px (d) in cells (*n* = 3 per group). ^∗^*P* < 0.05, ^∗∗^*P* < 0.01, ^∗∗∗^*P* < 0.001, and ^∗∗∗∗^*P* < 0.0001 vs. control; ^##^*P* < 0.01 and ^###^*P* < 0.001 vs. ani+CSE; ^&^*P* < 0.05 and ^&&&^*P* < 0.001 vs. CSE; ^$$^*P* < 0.01 and ani+SS-31+CSE vs. SS-31+CSE. Abbreviations: ani: anisomycin; CSE: cigarette smoke extract; GSH-Px: glutathione peroxidase; MDA: malondialdehyde; ROS: intracellular reactive oxygen species; SOD: superoxide dismutase.

ROS levels are an important indicator of intracellular oxidative stress. Anisomycin pretreatment reduced the suppressive effect of SS-31 on CSE-induced ROS production (Figure 10(a)) and abolished the downregulation of MDA activity and upregulation SOD activity in SS-31-treated BEAS-2B cells (Figures 10(b) and 10(c)), although it had no effect on GSH-Px activity (Figure 10(d)). These results indicate that inactivation of MAPK signaling is essential for the protective effect of SS-31—which may be specific to antioxidant enzymes—against oxidative stress induced by CSE.

### 3.10. SS-31 Prevents CSE-Induced Mitochondrial Dysfunction via Inactivation of MAPK Signaling

**Figure 11:**
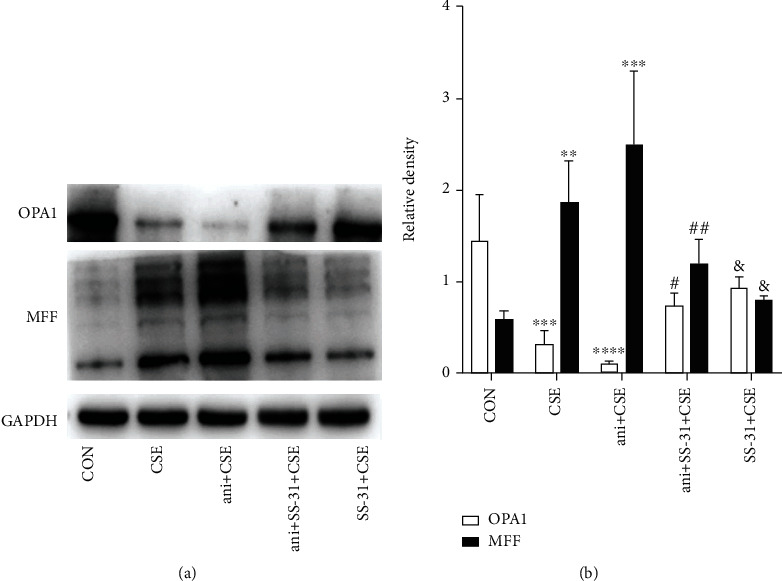
SS-31 protects against CSE-induced mitochondrial dysfunction via suppression of MAPK signaling. (a, b) Protein levels of OPA1 and MFF detected by western blot in BEAS-2B cells (*n* = 3 per group). ^∗∗^*P* < 0.01, ^∗∗∗^*P* < 0.001, and ^∗∗∗∗^*P* < 0.0001 vs. control; ^#^*P* < 0.05 and ^##^*P* < 0.01 vs. ani+CSE; ^&^*P* < 0.05 vs. CSE. Abbreviations: ani: anisomycin; CSE: cigarette smoke extract; MFF: mitochondrial fission factor; OPA1: optic atrophy 1.

The downregulation of OPA1 and upregulation of MFF induced by CSE in BEAS-2B cells were reversed by SS-31, but these changes were abolished by the MAPK activator anisomycin (Figures 11(a) and 11(b)). Thus, activation of the P38 MAPK signaling pathway can block the protective effects of SS-31 against CSE-induced mitochondrial dysfunction.

## 4. Discussion

SS-31 is a Szeto–Schiller peptide that selectively targets the inner mitochondrial membrane and exerts protective effects during inflammatory responses and oxidative stress [25–27]. In the current study, we found that SS-31 attenuated CS-induced airway inflammation, mucus hypersecretion, and oxidative stress in mice. SS-31 also preserved mitochondrial function through up- and downregulation of mitochondrial proteins (OPA1 and MFF, respectively) and by blocking the release of cytochrome c into the cytosol. The results of the RNA sequencing analysis suggested that these effects may be related to inhibition of CS-induced MAPK signaling. *In vitro* experiments demonstrated that SS-31 protected BEAS-2B cells from CSE-induced inflammation, oxidative damage, and mitochondrial dysfunction via suppression of the MAPK signaling pathway.

CS-induced mitochondrial oxidative stress amplifies airway inflammation and airway mucus hypersecretion and is closely associated with COPD progression [28, 29]. Antioxidants are beneficial in COPD as they inhibit the inflammatory response [30]. SS-31 was shown to suppress inflammation, as evidenced by the decreased levels of IL-6, IL-1*β* [16, 31], TNF-*α* [31], and MMP9 [14]. Additionally, SS-31 restored the activities of SOD, MDA [30], and MPA [12] and mitigated ROS production [32], thereby balancing oxidative status. These results provide novel evidence that SS-31 has a protective role against CS-induced airway inflammation and oxidative stress and thus has therapeutic potential for the treatment of CS-related lung disorders such as COPD.

CS-induced mitochondrial dysfunction has been linked to the initiation and progression of COPD [8], which may be effectively treated with mitochondrial-targeting antioxidants [28]. SS-31 restored the balance of cytochrome c levels between mitochondria and the cytoplasm in renal fibrosis [33] and obstructive nephropathy [34]. Pretreatment with SS-31 also reversed alterations in the expression of the mitochondrial proteins dynamin-related protein (DRP) 1, FIS1, mitofusin (MFN) 1, MFN2, and OPA1 in Alzheimer disease [35]. Here, we demonstrate for the first time that SS-31 counters CS-induced airway inflammation and oxidative stress by alleviating mitochondrial dysfunction.

The RNA sequencing analysis revealed that SS-31 altered the expression of 4038 genes in mouse lung. Interestingly, one of the genes that was upregulated was the circadian clock gene nuclear receptor subfamily 1 group D member (NR1D) 1. The downregulation of NR1D1 has been linked to pathologic changes in the respiratory tract induced by CS [36]. Pretreatment with an NR1D1 agonist and antagonist blocked IL-1*β* secretion and increased macrophage and neutrophil infiltration, respectively [37], while mutation of the NR1D1 gene enhanced inflammation and chemokine release [38, 39]. NR1D1 regulates mitochondrial energy production and enhances cellular antioxidant mechanisms to protect cells against oxidative stress [40]. Thus, the modulation of mitochondrial oxidative stress and inflammation by SS-31 may involve disruption of the circadian clock, with NR1D1 serving as a downstream target of SS-31.

The RNA sequencing results suggested that SS-31 regulates many aspects of cell function including epithelial cilium movement and cilium assembly. CS exposure was shown to result in cilia loss and impaired beating [41], and alterations in cilia structure or function have been implicated in COPD pathogenesis [42]. Signaling molecules involved in the occurrence and development of COPD such as MAPK and cAMP help regulate inflammation and airway remodeling, as does extracellular matrix degradation [43]. AMPK modulates inflammatory responses, senescence, mitochondrial dysfunction, and metabolic dysregulation [44]. There is increasing evidence that constitutive or aberrant MAPK activation contributes to several COPD-associated phenotypes including mucus overproduction and secretion, inflammation, and cytokine expression [45, 46]. Various small-molecule inhibitors may exert lung-protective effects by blocking the ERK1/2 and MAPK signaling [43, 47]. It was previously reported that SS-31 exerts antioxidant effects by suppressing the activation of P38 MAPK [48–50]. This was confirmed by our observation that SS-31 treatment reversed the increases in ERK and P38 phosphorylation in CS-exposed mice. Thus, SS-31 protects the lungs in COPD by attenuating CS-induced activation of the ERK/P38 MAPK pathway, which was supported by the finding that the MAPK activator anisomycin [51] partly reversed the anti-inflammatory and antioxidant effects of SS-31 in response to CSE in bronchial epithelial cells.

The current study had several limitations. Firstly, in order to minimize the number of mice used, we did not include a control group that was pretreated with SS-31 but was not exposed to CS. Secondly, two BALF samples were not lavaged successfully in the CS and SS-31 (high)+CS groups; because of the small sample size, the inhibitory effect of 5 mg/kg SS-31 on the number of neutrophils in the BALF may have been minimized. Thirdly, because of a lack of specific equipment, we did not examine the effect of SS-31 treatment on lung function in mice exposed to CS. Finally, the effects of anisomycin and SS-31 on mitochondrial cytochrome c were not evaluated because of the difficulty of extracting this protein. A major challenge of studies on CS-induced inflammation is translating the experimental data into clinically relevant studies. For the clinical application of SS-31 in the treatment of CS-related disorders in humans, additional studies are needed to determine the appropriate dosing and potential adverse effects.

## 5. Conclusion

The results of this study demonstrate that SS-31 has therapeutic potential for the treatment of CS-induced lung diseases—particularly COPD—based on its anti-inflammatory and antioxidant properties *in vitro* and *in vivo*, which involve the downregulation of MAPK signaling and modulation of mitochondrial function.

## Data Availability

Data used to support the findings of this study are available from the corresponding author upon request.

## Supplementary Materials

Supplementary Materials Supplementary Figure 1: correlation between MPO activity and the number of neutrophils in the lungs was analyzed. Supplementary Table 1: all western blot images with densitometry are summarized. Supplementary Table 2: the top 10 upregulated and downregulated DEGs identified by RNA sequencing analysis.
