# Supplementary material for: Mitochondrial-Targeting Antioxidant SS-31 Suppresses Airway Inflammation and Oxidative Stress Induced by Cigarette Smoke
Source: Oxid Med Cell Longev. 2021 Jun 15;2021:6644238. doi: 10.1155/2021/6644238 (PMC8219423; doi:10.1155/2021/6644238)
Supplement: Supplementary Materials — Supplementary Figure 1: correlation between MPO activity and the number of neutrophils in the lungs was analyzed. Supplementary Table 1: all western blot images with densitometry are summarized. Supplementary Table 2: the top 10 upregulated and downregulated DEGs identified by RNA sequencing analysis. [file 6644238.f1.zip › 6644238.f2.pdf]

Supplementary Table 1. All western blot images with densitometry

Figure 6 a)

Sample 1

OPA1

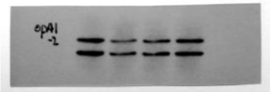

MFF

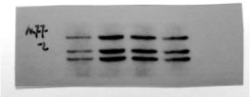

GAPDH

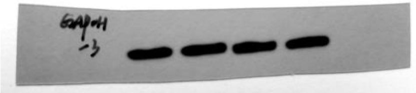

Sample 1

	CON	CS	SS-31(L)+CS	SS-31(H)+CS
OPA1	3523945	1693173	2326862	3051777
MFF	1670655	4302425	3851568	3122669
GAPDH	3104000	3149853	3008289	2983503
OPA1/GAPDH	1.135	0.538	0.773	1.023
MFF/GAPDH	0.538	1.366	1.280	1.047

Sample 2

OPA1

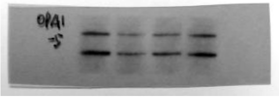

MFF

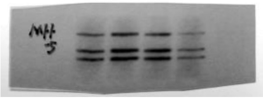

GAPDH

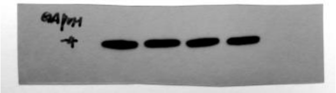

Sample 2

	CON	CS	SS-31(L)+CS	SS-31(H)+CS
OPA1	2134142	830241	1305763	1930246
MFF	3008866	4494431	3746897	1562962
GAPDH	2558558	2993834	2860858	3011834
OPA1/GAPDH	0.834	0.277	0.456	0.641
MFF/GAPDH	1.176	1.501	1.310	0.519

Sample 3

OPA1

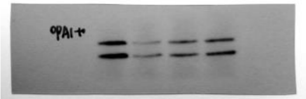

MFF

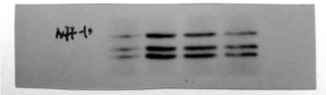

GAPDH

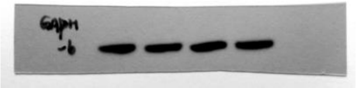

Sample 3

	CON	CS	SS-31(L)+CS	SS-31(H)+CS
OPA1	3549360	823512	1736434	2472032
MFF	1213271	4356659	3843423	2669702
GAPDH	2299195	2439821	2541556	2484954
OPA1/GAPDH	1.544	0.338	0.683	0.995
MFF/GAPDH	0.528	1.786	1.512	1.074

Sample 1

Figure 6 c) **Cyt(cytosol)**

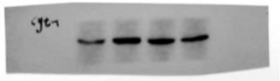

GAPDH

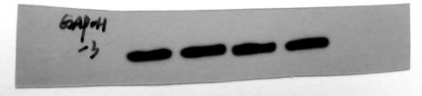

Figure 6 c)

Cyt(mit)

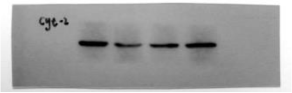

COXIV

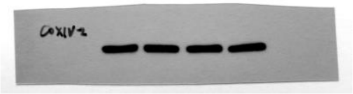

Sample 1

	CON	CS	SS-31(L)+CS	SS-31(H)+CS
Cyt(cytosol)	895711	1871451	1591284	1355533
GAPDH	3104000	3149853	3008289	2983503
Cyt(cytosol)/GAPDH	0.289	0.594	0.529	0.454
Cyt(mit)	2218775	1076273	1679449	2095660
COXIV	2521777	2552699	2572021	2699134
Cyt(mit)/COXIV	0.880	0.422	0.653	0.776

Sample 2

Cyt(cytosol)

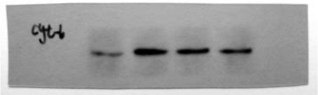

Figure 6 c) **GAPDH**

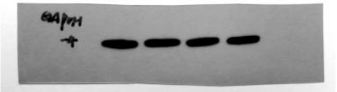

Cyt(mit)

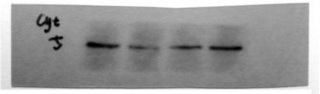

COXIV

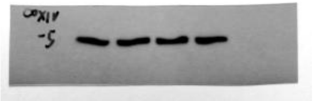

Sample 2

	CON	CS	SS-31(L)+CS	SS-31(H)+CS
Cyt(cytosol)	645515	1880270	1572932	1232011
GAPDH	2558558	2993834	2860858	3011834
Cyt(cytosol)/GAPDH	0.252	0.628	0.550	0.409
Cyt(mit)	1211147	370901	638844	1003984
COXIV	2137871	2203359	2099545	2061495
Cyt(mit)/COXIV	0.567	0.168	0.304	0.487

Sample 3

Cyt(cytosol)

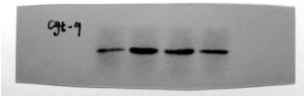

GAPDH

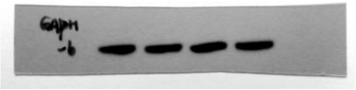

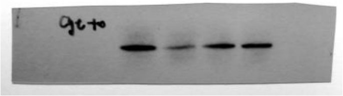

Cyt(mit)

COXIV

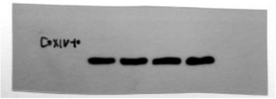

Sample 3

	CON	CS	SS-31(L)+CS	SS-31(H)+CS
Cyt(cytosol)	833344	1913228	1791164	1242140
GAPDH	2299195	2439821	2541556	2484954
Cyt(cytosol)/GAPDH	0.362	0.784	0.705	0.500
Cyt(mit)	2145813	622386	1443947	1546377
COXIV	2455719	2580462	2763621	2780949
Cyt(mit)/COXIV	0.874	0.241	0.522	0.556

Sample 1

Figure 8 a)

P-ERK

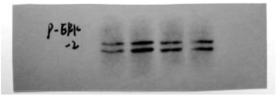

ERK

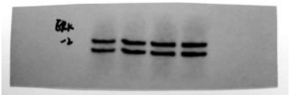

P-P38

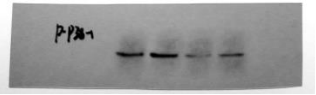

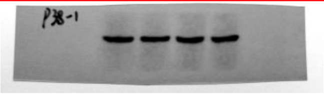

P38

Sample 1

	CON	CS	SS-31(L)+CS	SS-31(H)+CS
P-ERK	981306	2238003	1656092	1520395
ERK	2823049	3287956	3377976	3673276
P-ERK/ERK	0.348	0.681	0.490	0.414
P-P38	1069147	1544211	322272	590655
P38	2280716	2180264	2206065	2139871
P-P38/P38	0.469	0.708	0.146	0.276

Sample 2

P-ERK

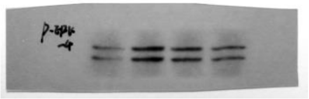

ERK

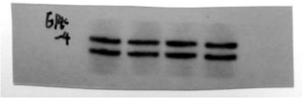

P-P38

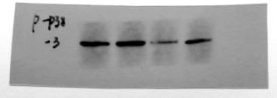

Figure 8 a)

P38

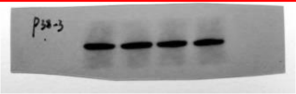

Sample 2

	CON	CS	SS-31(L)+CS	SS-31(H)+CS
P-ERK	951137	2804141	2135193	1600622
ERK	3481955	3273442	3635017	3848279
P-ERK/ERK	0.273	0.857	0.587	0.416
P-P38	1583949	1995573	647528	1193090
P38	2431833	2507356	2365148	2332376
P-P38/P38	0.651	0.796	0.274	0.512

Sample 3

P-ERK

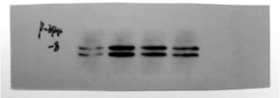

ERK

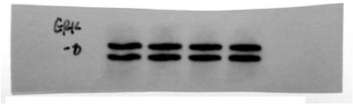

P-P38

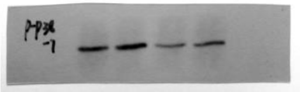

P38

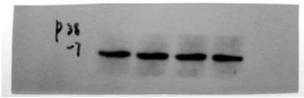

Sample 3

	CON	CS	SS-31(L)+CS	SS-31(H)+CS
P-ERK	810021	2141710	2032237	1853349
ERK	3364205	3092426	3009509	2800459
P-ERK/ERK	0.241	0.693	0.675	0.662
P-P38	1384150	1988653	689239	1021605
P38	2098943	2180177	2098699	2025590
P-P38/P38	0.659	0.912	0.328	0.504

Sample 1

P-P38

P38

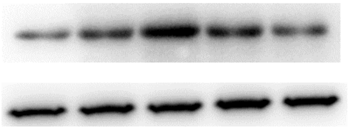

Sample 1

	CON	CSE	ani+CSE	ani+SS-31+CSE	SS-31+CSE
P-P38	132939	226080	355197	275667	196279
P38	248806	265576	260045	289179	265361
P-P38/P38	0.534	0.851	1.366	0.953	0.740

Figure 9 b)

Sample 2

P-P38

P38

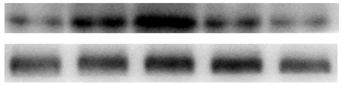

Sample 2

	CON	CSE	ani+CSE	ani+SS-31+CSE	SS-31+CSE
P-P38	196734	321413	405276	259375	184133
P38	259538	282502	318757	315403	230865
P-P38/P38	0.758	1.138	1.271	0.822	0.798

Sample 3

P-P38

P38

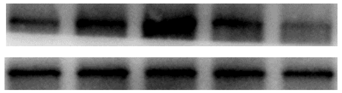

Sample 3

	CON	CSE	ani+CSE	ani+SS-31+CSE	SS-31+CSE
P-P38	231843	292806	364752	296227	200600
P38	298335	278072	317362	292265	239817
P-P38/P38	0.777	1.053	1.149	1.014	0.836

Figure 11 a) **Sample 1**

OPA1

MFF

GAPDH

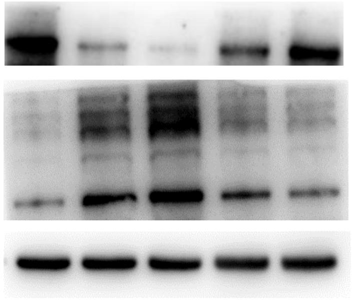

Sample 1

	CON	CSE	ani+CSE	ani+SS-31+CSE	SS-31+CSE
OPA1	389430	77540	32619	210804	311789
MFF	179762	672237	1005168	433782	294981
GAPDH	344342	358677	331555	323634	346481
OPA1/GAPDH	1.131	0.216	0.098	0.651	0.900
MFF/GAPDH	0.522	1.874	3.032	1.340	0.851

Sample 2

OPA1

MFF

GAPDH

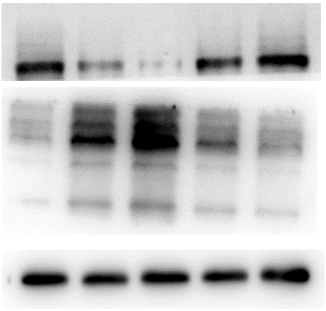

Sample 2

	CON	CSE	ani+CSE	ani+SS-31+CSE	SS-31+CSE
OPA1	393739	147048	54211	264023	398435
MFF	196150	696592	994968	403667	281289
GAPDH	340292	301032	346786	294935	370611
OPA1/GAPDH	1.157	0.488	0.156	0.895	1.075
MFF/GAPDH	0.576	2.314	2.869	1.369	0.759

Sample 3

OPA1

MFF

GAPDH

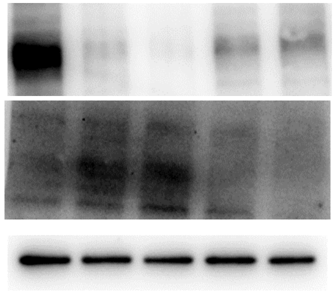

Sample 3

	CON	CSE	ani+CSE	ani+SS-31+CSE	SS-31+CSE
OPA1	683186	80710	25419	201966	210487
MFF	233370	391827	408495	260171	199566
GAPDH	335563	276272	260049	289540	248721
OPA1/GAPDH	2.036	0.292	0.098	0.698	0.846
MFF/GAPDH	0.695	1.418	1.571	0.899	0.802
