# Supplementary material for: Mitochondrial-Targeting Antioxidant SS-31 Suppresses Airway Inflammation and Oxidative Stress Induced by Cigarette Smoke
Source: Oxid Med Cell Longev. 2021 Jun 15;2021:6644238. doi: 10.1155/2021/6644238 (PMC8219423; doi:10.1155/2021/6644238)
Supplement: Supplementary Materials — Supplementary Figure 1: correlation between MPO activity and the number of neutrophils in the lungs was analyzed. Supplementary Table 1: all western blot images with densitometry are summarized. Supplementary Table 2: the top 10 upregulated and downregulated DEGs identified by RNA sequencing analysis. [file 6644238.f1.zip › 6644238.f3.pdf]

Supplementary Table 2. The top 10 up- and down-DEGs

Up DEGs	Down DEGs
Dbp	Has1
Nr1d2	Cd5l
E030044B06Rik	Arntl
Per3	Adamts4
Cxcr6	Fam107a
Nr1d1	Spon2
Plk5	Cdsn
Ky	Fam124b
Ccna1	Gm11827
Six4	Npas2
