# Supplementary figures and images for: Mitochondrial-Targeting Antioxidant SS-31 Suppresses Airway Inflammation and Oxidative Stress Induced by Cigarette Smoke
Source: Oxid Med Cell Longev. 2021 Jun 15;2021:6644238. doi: 10.1155/2021/6644238 (PMC8219423; doi:10.1155/2021/6644238)

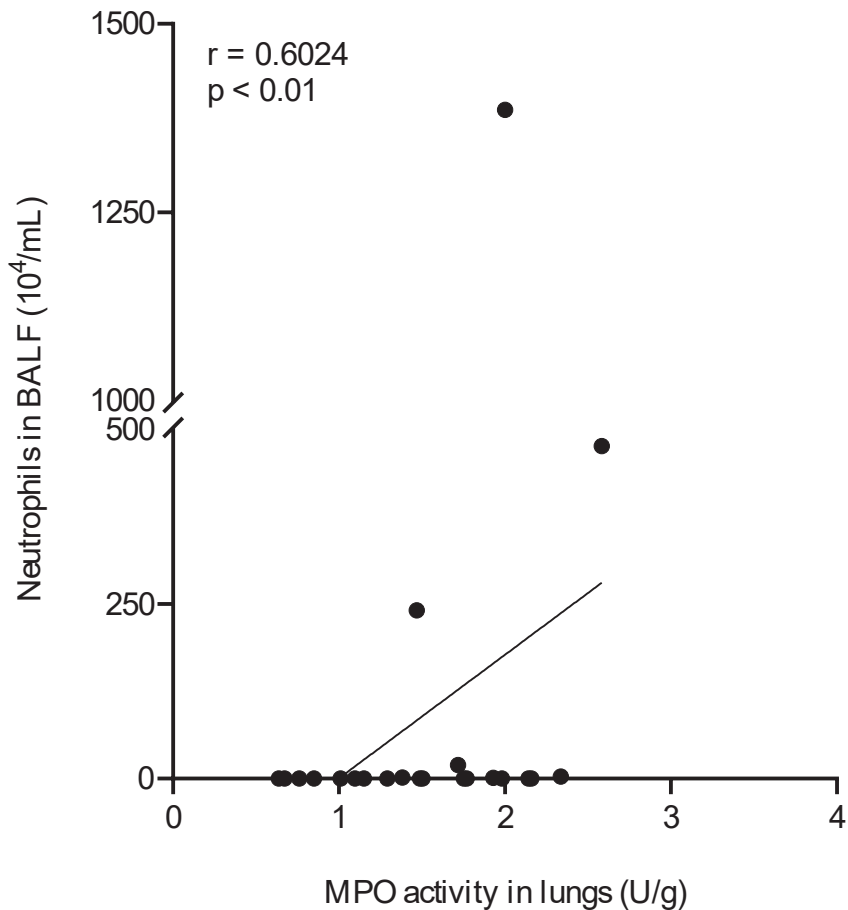

Supplement: Supplementary Materials — Supplementary Figure 1: correlation between MPO activity and the number of neutrophils in the lungs was analyzed. Supplementary Table 1: all western blot images with densitometry are summarized. Supplementary Table 2: the top 10 upregulated and downregulated DEGs identified by RNA sequencing analysis. [file 6644238.f1.zip › 6644238.f1.pdf]
